# Androstenedione (a Natural Steroid and a Drug Supplement): A Comprehensive Review of Its Consumption, Metabolism, Health Effects, and Toxicity with Sex Differences

**DOI:** 10.3390/molecules26206210

**Published:** 2021-10-14

**Authors:** Marwa T. Badawy, Mansour Sobeh, Jianbo Xiao, Mohamed A. Farag

**Affiliations:** 1 Department of Biology, School of Sciences and Engineering, The American University in Cairo, New Cairo 11835, Egypt; marwa-tawfik@aucegypt.edu; 2 AgroBioSciences, Mohammed VI Polytechnic University, Lot 660, Hay Moulay Rachid, Ben-Guerir 43150, Morocco; 3 International Research Center for Food Nutrition and Safety, Jiangsu University, Zhenjiang 212013, China; jianboxiao@uvigo.es; 4 Department of Analytical Chemistry and Food Science, Faculty of Food Science and Technology, University of Vigo, E-36310 Vigo, Spain; 5 Pharmacognosy Department, College of Pharmacy, Cairo University, Kasr el Aini St., Cairo P.B. 11562, Egypt; 6 Chemistry Department, School of Sciences Engineering, The American University in Cairo, New Cairo 11835, Egypt

**Keywords:** androstenedione, health effects, toxicity, pharmacokinetics, metabolism, drug interaction, pharmacogenetics

## Abstract

Androstenedione is a steroidal hormone produced in male and female gonads, as well as in the adrenal glands, and it is known for its key role in the production of estrogen and testosterone. Androstenedione is also sold as an oral supplement, that is being utilized to increase testosterone levels. Simply known as “andro” by athletes, it is commonly touted as a natural alternative to anabolic steroids. By boosting testosterone levels, it is thought to be an enhancer for athletic performance, build body muscles, reduce fats, increase energy, maintain healthy RBCs, and increase sexual performance. Nevertheless, several of these effects are not yet scientifically proven. Though commonly used as a supplement for body building, it is listed among performance-enhancing drugs (PEDs) which is banned by the World Anti-Doping Agency, as well as the International Olympic Committee. This review focuses on the action mechanism behind androstenedione’s health effects, and further side effects including clinical features, populations at risk, pharmacokinetics, metabolism, and toxicokinetics. A review of androstenedione regulation in drug doping is also presented.

## 1. Introduction and Occurrence

Steroidal hormones are the key players in the stress response and reproduction in mammals. Consequently, they have been considered health biomarkers in both land and aquatic wildlife [1]. Androstenedione or 4-Androstene-3-17-dione (4A) is a naturally occurring steroidal hormone produced in both sexes from the gonads and adrenal glands [2] and serving as an intermediate in the biosynthesis of testosterone [3]. It is produced mainly at normal conditions by the adrenals (2–3 mg/day) and testis (0.5 mg/day). Bone is the target organ of androgens, which affect both the maturation of bones as well as the homeostasis of mature bones. Androgen deficiency is linked to premature bone loss [4] and with the elevated frequency of osteoporotic features in men [5]. The treatment of postmenopausal women with anabolic steroids favorably impacts the bone mass [6]. Androgens stimulate human and murine osteoblast-like cells proliferation in vitro [7]. Androgen receptors are also characterized in cultured human osteoblast-like cells [8], suggesting that they can act directly on bone cells via a receptor-mediated mechanism [9].

Currently, androstenedione is frequently administered to bodybuilders and athletes [10], due to its effect as an immediate precursor to testosterone in the androgens’ intrinsic synthetic pathways [11]. Androstenedione is sold without medical prescription in the United States and the sport supplementation market registered annual revenue of 1.4 billion in 1999 [], with expectation to continue growing [12]. The administration of androstenedione as a supplement has become more popular among athletes, especially after the MLB player Mark McGuire admitted using it during his historic 1998 chase of Roger Maris’s single-season home run record [13]. Consequently, androstenedione is sold to athletes as a potential anabolic agent [12], and the sport supplement industry is profiting some multimillion dollars per year.

Nutraceuticals are used as dietary supplements to be used for the treatment or prevention of several diseases [14]. Androstenedione is not typically considered among nutraceuticals or regulated by the Food and Drug Administration (FDA) owing to the ongoing debate about its therapeutic effectiveness for preventing or treating certain chronic diseases such as anxiety and improving mental acuity [15]. Nowadays, licensed healthcare professionals and physicians often prescribe dietary supplements of androstenedione to counteract the effects of age-related muscle loss (sarcopenia) to improve lifespan as well as quality of life in older people [16]. However, the use of androstenedione in some individuals, including athletes, can cause an increase in the testosterone to epitestosterone ratio (T/E) above the International Olympic Committee (IOC) cut-off of 6 [17,18], which is likely to occur in men who take testosterone [19]. The reason behind the increase in the T/E ratio is an increase in urinary excretion of testosterone concurrent with a decrease in urinary excretion of epitestosterone [12].

**Figure 1:**
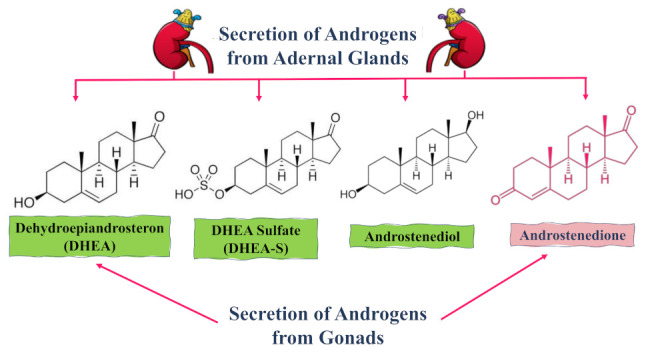
Diagram showing the secretion origins of the main androgenic hormones (steroids) from both adrenal glands and gonads.

Several androgens are secreted by the endocrine glands, including androstenedione, 5-androstene-3b,17b-diol (androstenediol), dehydroepiandrosterone sulfate (DHEAS), and dehydroepiandrosterone (DHEA) [20]; see Figure 1. The androgenic hormones, dehydroepiandrosterone (DHEA) and androstenedione, are produced by the adrenal glands and act as precursors in the estrogen and testosterone hormones production [21,22]. Their production peaks during the mid-20s and then starts to decline in a controlled manner after the third decade of life [23].

## 2. Methods

To conduct this review, an electronic search was performed via the PubMed, SciFinder, Scopus, Google Scholar, Web of Science, and Science Direct search engines. The keywords used were “Androstenedione; health effects; toxicity; pharmacokinetics; metabolism; drug interaction; pharmacogenetics”. Both review articles and original studies focusing on the topic of the review were included from 1976 to 2020. We excluded papers for which only an abstract was found, preprints, and conference proceedings. Androstenedione toxicity was reported only from the oral administration not from the indigenous hormone.

## 3. Distribution in Nature

**Figure 2:**
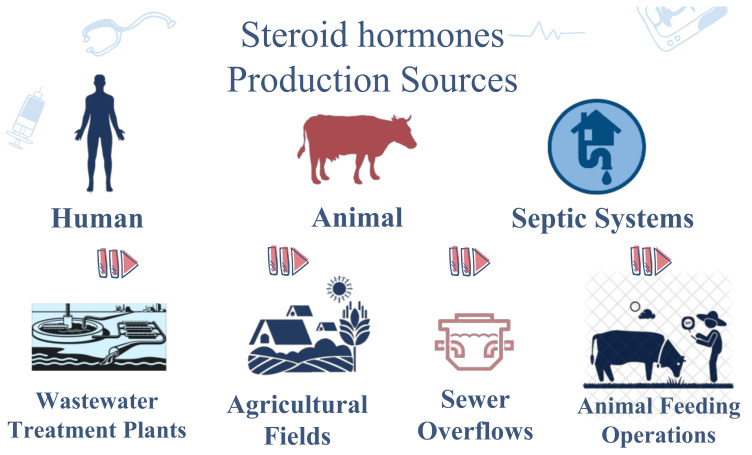
Steroid hormones’ various origins in nature, including human and animal sources, septic systems, concentrated animal feeding operations, combined sewer overflows, wastewater treatment plants, and agricultural fields.

Steroidal hormones in nature, as illustrated in Figure 2, can be found in human and animal sources, septic systems, concentrated animal feeding operations, combined sewer overflows, wastewater treatment plants, and agricultural fields [24,25,26]. Once steroid hormones are present in surface water, they are subjected to several biotransformation and removal processes, such as sorption to sediments or colloids, direct or indirect photo-degradation, and biodegradation [27,28,29].

Androstenedione was also found in the Fenholloway River water column, likely derived from the microbial degradation of several phytosteroids, such as stigmastanol, *β*-sitosterol, and campesterol. For instance, the microbial conversion of phytosterols is being widely applied for the production of steroidal drug intermediates such as AD and 9-OHAD (9-hydroxy-4-androstene-3,17-dione) [30,31,32]. These phytosteroids are found in wastes created from the conversion of pine tree into pulp [33]. 

In aquatic habitats, where there is huge effluent release from livestock farms, national sewage treatment plants, and pulp and paper factories [34], elevated levels of androstenedione were detected in Beijing in the sewage from seven different wastewater treatment plants at levels ranging from 5 ng/L to 270 ng/L [35]. It was also detected in Guangdong Province in two other different types of wastewater treatment plants at mean levels of (1368 ± 283) ng/L [36], and in natural waterways at levels up to 480 ng/L in Japan [37]. These residues of androstenedione could cause substantial risks to aquatic organisms as well as humans upon further exposure. Previous studies have described the adverse effect of androstenediones on the aquatic systems. For instance, laboratory and field studies discovered the masculinization of *Gambusia holbrooki,* the female mosquitofish, living in water and contaminated by pulp mills [38], specifically with androstenedione and androstadienedione (ADD) [34,39]. Furthermore, Hou [40] explored the endocrine disruption induced by androstenedione over the growth cycle of zebrafish embryos. The authors reported that the levels of mRNA of some primary genes (Lhb, Hsd20b, Star, Cyp19a, Fshb, Vtg1, Pomc), which participate in the receptor signaling pathways, were altered after exposure to androstenedione, suggesting that androstenedione’s adverse effects are observed throughout the growth of zebrafish embryos [40]. Whether such changes in gene levels could be also observed in humans following ingestion of these contaminated fish have yet to be reported.

The purpose of this review is to provide insights into and critical analysis of androstenedione as a natural hormone, along with its different metabolic reactions. Extensive knowledge regarding androstenedione consumption, action mechanisms for its health benefits and side effects, in addition to its pharmaco/toxicokinetics and clinical features is presented for the first time.

## 4. Androstenedione Distribution and Metabolism

### 4.1. Absorption and Distribution

**Figure 3:**
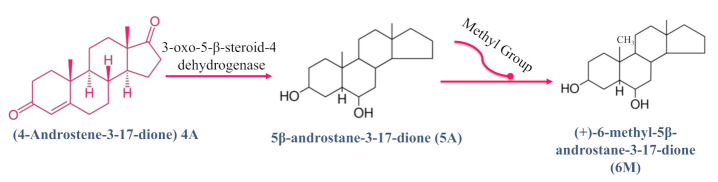
The chemical equation of the enzyme-catalyzed reaction of 4-androstene-3-17-dione (4A) hormone with 3-oxo-5-*β*-steroid-4- dehydrogenase enzyme to produce 5*β*-Androstane-3-17-dione (5A), followed by the production of (+)-6-methyl-5*β*-androstane-3-17-dione (6M) with a methyl group attached at the C6 position [41].

**Figure 4:**
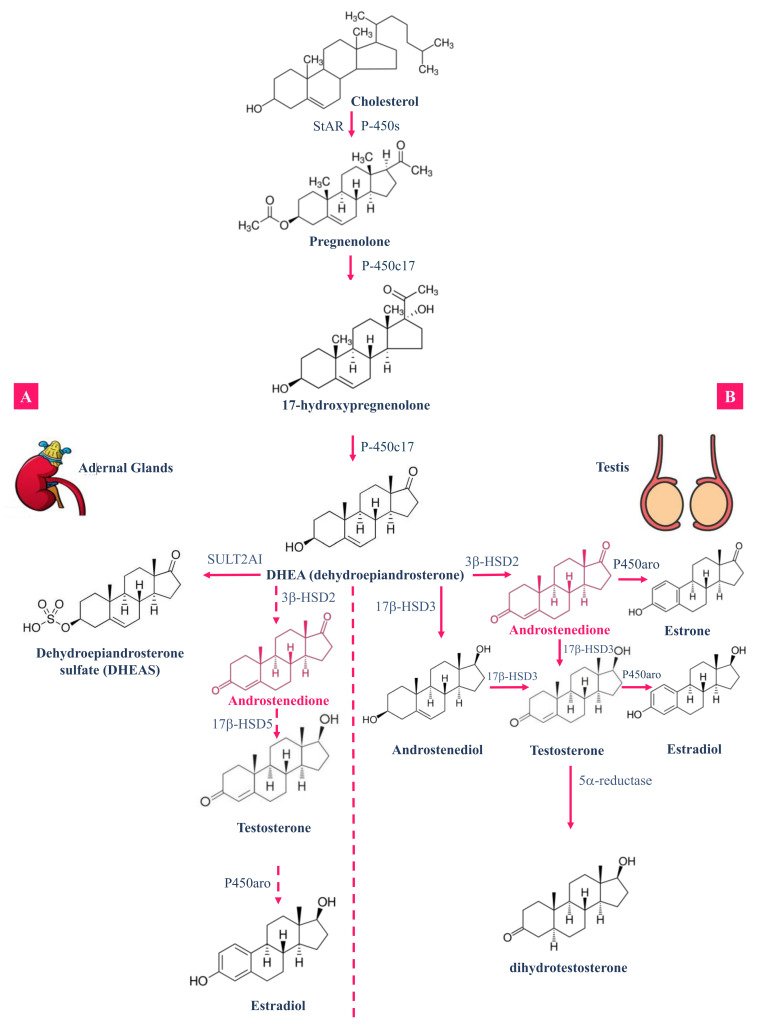
Production of steroidal hormones from cholesterol, including androstenedione production by the enzyme 3ß-HSD2 in both adrenal glands (**A**) and testis (**B**), as well as the production other essential androgens throughout the enzymatic reaction. Androstenedione is highlighted in pink in both cycles. Enzyme codes are: Steroidogenic Acute Regulatory protein (StAR), Cytochromes P450, Sulfotransferase (SULTs), 3-*β* hydroxysteroid dehydrogenase (3*β*-HSD), and aromatase cytochrome P450 (P450aro).

The study of androstenedione and its derivatives’ interaction with plasma proteins is of value to provide insight into the bioavailability and distribution of these specific molecules [41], with various biological actions. Plasma proteins typically bind reversibly to the hydrophobic hormones, drugs, and metabolites and are circulating in the bloodstream to extend their clearance time from the body. The importance of the binding affinity of plasma proteins lies in the fact that it can help to determine free and effective concentrations of drugs, and further regulate their pharmacodynamic and pharmacokinetic properties [42]. Lipoproteins, α1-acid glycoprotein (AGP) and human serum albumin (HSA) are the major carriers of proteins in blood plasma [42]. As mentioned earlier, the natural steroid hormone, 4-androstene-3-17-dione (4A), is produced in the adrenal glands and gonads. Physiologically, 4A, in the existence of 3-oxo-5-*β*-steroid-4- dehydrogenase, is converted into the 5*β*-androstane-3-17-dione (5A) steroid molecule. On the other hand, (+)-6-methyl-5*β*-androstane-3-17-dione (6M) is another steroidal hormone derivative where a methyl group is connected to the C6 position of 5*β*-androstane-3-17-dione [41], as illustrated in Figure 3. Moreover, 4A is also an immediate precursor for testosterone via the action of 17-*β*-hydroxysteroid dehydrogenase [43]; see Figure 4A,B.

Nerusu [41] and his colleagues reported that 6M, 4A, and 5A steroid molecules can form stable complexes with AGP and HSA, with higher binding affinity to HSA than AGP [41]. The binding constants obtained for 6M, 4A, and 5A with HAS were (9.5 ± 0.2) × 10^4^, (5.3 ± 2) × 10^4^, and (5.3 ± 1) × 10^4^ M^−1^, respectively, compared to (2.1 ± 0.7) × (10^4^, 7.4 ± 4) × 10^3^, and (2.6 ± 0.6) × 10^3^ M^−1^ with AGP for 6M, 4A, and 5A, respectively [41]. Consequently, HAS-androstenedione and its derivative complexes were stabilized after 15 ns and found to maintain their stable structures [41].

### 4.2. Metabolism

Sometimes termed androstenedione, 4-Androstene-3-17-dione (4A) is considered the key intermediate in steroid metabolism [41]. The metabolism of androstenedione in the different biological systems is presented in the next subsections.

#### 4.2.1. Metabolism in Fungi

One of the most well-studied biotransformation of testosterone, androstenedione, and progesterone derivatives was carried out in a cultured fungi strain of *Absidia coerulea* [44]. All of the examined substrates were transformed or hydroxylated, which enabled researchers to isolate biotransformed metabolites [44]. The same author and his colleagues studied the transformation of testosterone, androstenedione, and progesterone derivatives with an extra C1-C2 double bond and/or 17 α-methyl group in a cultured fungi strain of *Absidia coerulea* as a model to assess the eukaryotic biotransformation of steroids, including 4A. Although the substrates included the 4-ene-3-oxo system, they displayed some differences in the substituents at C-17 and/or the existence of the additional C1-C2 double bond.

#### 4.2.2. Metabolism in Humans and Non-Human Primates

Firstly, 11-oxygenetad steroids circulate in the body with different serum levels, varying among species, with the highest levels detected in humans and non-human primates [45]. The major chemicals in the synthesis of steroid drugs are known as 4-androstene-3,17-dione (androstenedione, AD) and 1,4-androstadiene-3,17-dione (androstadienedione, ADD). One of the crucial physiological mechanisms in mammalian organisms is steroid hydroxylation because of its role in pro-drug activation or the detoxification of exogenous steroids [46]. The formation of androgenic products is based on the availability of the needed substrates [47]. For instance, androstanedione was reported to be the principal metabolite of androstenedione in human fetal epiphyseal cartilage [48] and in human hair roots [49]. Androstenedione can be synthesized from dehydroepiandrosterone and further converted into either testosterone through the action of 17*β*- hydroxysteroid dehydrogenase, or to estrone via the aromatase enzyme complex [50]. DHEA is converted into androstenedione in the adrenal cortex, where it can be either aromatized to estrone or de-hydrogenated in the liver to yield testosterone [51]. Gene polymorphisms that encode for key enzymes involved in such pathways could have a modulation effect on the endogenous hormone levels; hence, it can influence the risk of hormone-related cancer diseases [52,53], and it can account for different responses to steroids in humans.

It is worth mentioning that the metabolism of androgens, in vivo, in bone could be more challenging than under in vitro conditions due to the hormone regulation of enzyme activities [9]. Androgens are circulated through the peripheral tissues in the body, which is followed by immense metabolism and subsequent excretion in urine [54]. Metabolism of androgen takes place mainly via hydroxysteroid dehydrogenases, reductases, and conjugation enzymes [54]. Additionally, progesterone is biosynthesized from pregnenolone as an intermediate product of androstenedione and testosterone biosynthesis in the testicles, as well as in the adrenal cortex to a certain extent [55]. On the other hand, testosterone is converted in an extensive manner into androstenedione, and only a very small percentage of the testosterone that is assembled in the body is metabolized to testosterone glucuronide and detected in urine [54]. Moreover, DHEA and androstenedione are metabolized primarily to androsterone etiocholanolone and androsterone [54]. Androstenedione in the blood can turn into testosterone and further to estradiol or 5α-dihydrotestosterone (DHT) in peripheral tissues. In other cases, androstenedione can be converted into estrone and then estradiol without testosterone formation [55]; see Figure 4B. Moreover, androstenedione can be metabolized and converted into several potent estrogens or androgens, such as estradiol, estrone, or testosterone [56], as illustrated in Figure 4A.

#### 4.2.3. Metabolism in Animals and Rodents

**Figure 5:**
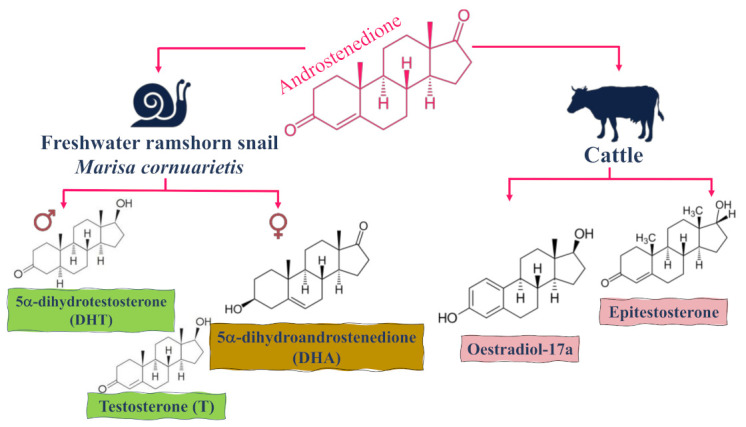
The main identified metabolites of androstenedione in cattle and the freshwater ramshorn snail *Marisa cornuarietis* in both males and females.

The main identified metabolites of androstenedione in cattle are oestradiol-17 and epitestosterone [57]—see Figure 5—whereas the main metabolite in sheep is the 17-epimer [58]. Furthermore, 3(17)-hydroxysteroid dehydrogenase (HSD) enzyme, which has been detected in kidney and liver tissues from hamsters and rabbits, is a possible candidate involved in the conversion of androstenedione to epitestosterone [59]. Moreover, 17-HSD enzyme has been isolated and characterized from an intestinal *Eubacterium sp*. [60], suggesting that colon bacteria could also play a role in androstenedione metabolism. The effect of the gut microbiota on the metabolism of androstenedione and its different analogues is less well-understood, especially given the inter-individual variability of gut composition, and should be examined in the future.

Valdés [61] studied the oxidative metabolism of androstenedione in rats during starvation and in a streptozotocin (STZ) animal model of diabetes in order to detect any similarities or differences that could be observed under these two conditions. Results revealed that the ketotic conditions in both cases of diabetes and fasting animals were accompanied by higher levels of free fatty acids or their metabolites [61]. This was associated with distinct alterations in the metabolic activities, which is specific to individual cytochrome P450 associated with androstenedione metabolism; see Figure 4 [61].

Androstenedione has been identified in various invertebrate (animals without backbones) species and tissues [62]. Vertebrates and other invertebrates are known for their ability to metabolize androstenedione to testosterone (T) via a 17*β*-hydroxysteroid dehydrogenase (17*β*-HSD)-catalyzed pathway [63]. In invertebrates, microsomal fractions obtained from the digestive gland/gonad complex of *Marisa cornuarietis,* the freshwater ramshorn snail, revealed that androstenedione is largely converted to 5α-dihydrotestosterone (DHT) and testosterone (T) in males versus to 5α-dihydroandrostenedione (DHA) in females, as shown in Figure 5 [64]. Furthermore, the ratio of 17-reduced metabolites (DHT and T) versus DHA was higher in males (3–42) than that detected in females (0.13–0.78) in all cases and regardless of the sampling time [65]. More importantly, the conversion of testosterone into epitestosterone by androstenedione is highly unlikely to occur as many attempts to illustrate this pathway have failed [46]. However, other potential pathways could exist and have yet to be confirmed [12].

## 5. Hormonal Effects of Androstenedione AD (or 4A)

Androstenedione (androst-4-ene-3,17-dione) is an intermediate endogenous androgen that leads to the biosynthesis of testosterone, in addition to its exogenous administration in dietary supplements [66]. In this section, we review effects reported for endogenous androstenedione as well as exogenously administered in dietary supplements in both sexes.

### 5.1. Hormonal Effects of AD in Males

The effect of androstenedione supplementation on exercising and basal testosterone levels has been extensively reported from supplementation at dosages of 100–200 mg of androstenedione in humans [10,67]. A study conducted by Wallace [68] reported a basal testosterone level of 6.1 ng/mL for the participants with a mean age of (48.1 ± 3.9) years after the administration of androstenedione (100 mg) for a 12 weeks. To support these findings, another study reported by Leder [10] revealed that 100 mg supplementation of androstenedione per day for 7 days was not sufficient to raise testosterone levels in men, whereas a significant increase in testosterone levels was observed at a 300 mg dose level to reach in participants testosterone levels of 4.93 ng/mL, with a mean age of 31.5 years [10]. Such a result was in accordance with a previous report in which an oral 300 mg dose of androstenedione for 7 days showed an increase in testosterone levels, suggesting that short-term supplementation of androstenedione at 300 mg can significantly increase testosterone responses in older men [69].

### 5.2. Hormonal Effects of AD in Females

A study was conducted at the same dosage of 100 mg androstenedione (single dose, oral) in healthy postmenopausal women by Leder [70] in which they explored the effects of androstenedione on the serum levels sex steroids. After the administration of androstenedione (100 mg), serum levels of estrone were increased by up to 115% over 12 h in comparison to the control, versus a 450% increase in testosterone level over 12 h at the same dosage relative to the control [70]. Whether such a large increase in testosterone levels in women could lead to androgenic effects has yet to be examined. Similar results were observed by Kicman [71] from a single dose androstenedione (100 mg) on plasma testosterone over 24 h in 10 healthy young ladies at ca.16-fold greater than the control group. Such observed testosterone levels were comparable to those noticed in the misuse of testosterone for anabolic purposes, suggesting deleterious side effects of the modified sex hormone levels in women receiving androstenedione [71]. Other reports have supported these earlier observations in young women after the administration of androstenedione (100–300 mg, single dose) leading to increase in both serum estradiol (1.5–2 times) and serum testosterone by (5–10 times) at 4 h after dose administration [72], confirming a much greater increase in the male sex hormone than female sex hormones with androstenedione administration. In other words, the ingestion of androstenedione (100 or 200 mg) does not alter serum testosterone concentrations in young men [10,67], while ingesting of a single dose of 100–300 mg androstenedione leads to a 34% increase in the levels of serum total testosterone that lasts for 4–6 h [10].

### 5.3. Hormonal Effects of AD in Children

Children with obesity suffer from higher levels of adrenal androgen than their normal counterparts, which could lead to accelerated pre-pubertal growth in these children [73]. Kwon [74] and his colleagues examined the association between bone age (BA) and chronological age (CA) ratio (BA/CA) and androstenedione and testosterone levels. The effects of androgens or their metabolites on the skeleton are employed by direct stimulation of the estrogen receptor (ER) α, ER β and androgen receptor (AR). The expression of the androgen receptor (AR) was measured at several ages with no major sex difference in the human growth plate chondrocytes. As a result, androgens such as androstenedione may have an essential role in bone maturation in pre-pubertal children of both sexes [75].

### 5.4. Hormonal Effects in Female Rats

Hormone replacement therapy is a potential strategy for the protection of bones from postmenopausal osteoporosis [76]. However, there are multiple disadvantages due to their potential harmful side effects in other organs [77]. It is unclear whether androstenedione could impact the levels of physiological hormones by changing the liver enzyme activities that metabolize steroid hormones or not. Hence, Flynn [78] conducted a study on mature female rats, where they were gavaged with androstenedione (0, 5, 30, or 60 mg/kg/day) two weeks before mating and continued through gestation day 19. In addition, non-pregnant female rats were gavaged for the same timeframe with androstenedione (0 or 60 mg/kg/day) and the liver was further dissected from pregnant rats on gestation day 20, as well as from non-pregnant rats after five weeks of treatment [78]. Liver microsomes were incubated with testosterone, leading to the production of 6-hydroxytestosterone, 15-hydroxytestosterone, 7- hydroxytestosterone, 16- hydroxytestosterone, and 2-hydroxytestosterone at high levels compared to controls. The formation of 6-hydroxytestosterone was observed at both 30 and 60 mg/kg/day dose levels. On the other hand, in non-pregnant rats, androstenedione (60 mg/kg/day) markedly increased the formation of 15-, 6-, 16 -, and 2 -hydroxytestosterone [78]. The results highlighted that high oral doses of androstenedione could enhance some female rat liver cytochrome P450 activities that metabolize steroid hormones [78,79]. It is worth noting that there were no response differences among androstenedione doses between pregnant and non-pregnant female rats [78].

## 6. Receptors of Androstenedione

Steroid receptors nowadays are receiving increasing attention, considered to be responsible for many regulatory complexities within the vertebrate lineage [80,81]. Moreover, the nuclear receptor superfamily is an ancient lineage of transcription factors that are activated by small ligands [82]. It is also hypothesized that nuclear steroid receptors emerged at or before the radiation of vertebrates. However, the androgen receptor (Ar) evolved only in the gnathostome line [83]. The androgen-Ar complex was found in the extant jawless vertebrate male sea lamprey (Petromyzon marinus) [83]. The detected androgen with the highest affinity was androstenedione (Ad).

A study was conducted by Zucker [84] to explore the effects of androstenedione on thromboxane A2 (TXA2) receptor expression in rat aortic smooth muscle (RASM) cells and Human Erythroleukemia (HEL) cells [84]. The latter have been used as a model of megakaryocytes, which is the progenitor cell for platelets [85]. Both cell lines were incubated with androstenedione (250, 500, or 750 nM), testosterone (150 nM), or the vehicle for a period of 48 h. The results revealed that androstenedione is capable of increasing the number of TXA2 receptors in HEL cells, as well as in RASM cells, significantly, with no significant change in affinity [84].

## 7. Androstenedione Doping

Androstenedione encompasses potential health risks as a typical steroid, especially if it is abused [86]. Consequently, the US Food and Drug Administration mandated that androstenedione-contained products should not be marketed or distributed as other dietary supplements [87].

The use of over 100 doping agents is prohibited by the Medical Commission of the International Olympic Committee (IOC) for athletes and these agents are classified into five categories including anabolic agents [88]. Several athletes, however, use different types of anabolic–androgenic steroids (AAS), from synthetic anabolic steroids (i.e., stanozolol) to natural hormones (i.e., testosterone), administered by injection in order to increase their muscular mass and to upgrade their performance in training sessions [89]. The misuse of these restricted anabolic steroids is controlled by assessing the existence of the banned substances or any of their biotransformed metabolites in urine samples from athletes [90]. An androstenedione German patent reported that the ingestion of androstenedione by young individuals can lead to an increase in serum levels of testosterone by ca. 237% within 15 min, and this is followed by a secondary increase of 48–97% within 3 to 4 d [91]. Such increased levels in serum testosterone appear to represent solid responses to this dietary supplementation, while it has been reported on the World Anti-Doping banned list [92].

To assess the metabolism of anabolic steroids in humans, Lévesque [93] and his colleagues studied androstenedione and norandrostenedione to evaluate their in vitro incubations. These two steroids are precursors of testosterone and nortestosterone, respectively, and they are banned by the Medical Commission of the IOC as well [88].

## 8. Androstenedione Toxicity

The weak androgen, androstenedione, was found to be converted to stronger carcinogenic estrogens or androgens, including estradiol estrone or testosterone [94]. A limited number of studies have investigated the carcinogenic or tumor-promoting activities of androstenedione. As early as 1957, Bischoff [95] and his team reported that androstenedione increased the number of fibrosarcomas over 18 months, tested as a by-product, in male Marsh-Buffalo mice when subcutaneously injected for one to two times per 2-month period in a dose of 15 mg/mouse. Another study by Dauvois [96] reported that androstenedione administration increased the tumor size in ovariectomized rats when the level of androstenedione was kept at 500 µg (similar to those found in the circulation of post-menopausal women) and this was more attributed to the conversion of androstenedione to estradiol. Co-administration of an aromatase inhibitor blocked the former effect. Additionally, dehydroepiandrosterone (DHEA, androstenedione precursor) enhanced hepatocellular carcinomas, induced by hepatocarcinogen aflatoxin B1, in rats and rainbow trout at a daily dosage of 222 ppm, which corresponds to half of that of the dose administered to humans in clinical trials [97,98].

In contrast, the administration of androstenedione at three dose levels, namely 5, 30, or 60 mg/kg body weight/day, in pregnant rats initiated 2 weeks before mating and continued till day 19 of gestation revealed no significant differences in biomarkers of hepatotoxicity, including aspartate aminotransferase, serum alanine aminotransferase, glutathione, and glutathione S-transferase, lipid peroxidation, lactate dehydrogenase, total microsomal P450, and nuclear DNA damage on gestation day 20 [99]. Another study by Wieczerzak [100] demonstrated that androstenedione showed no effect on serum total triglycerides, cholesterol, or HDL-cholesterol, suggesting that it does not affect the lipid profile, but it significantly decreased prostaglandin E2 in pregnant and non-pregnant rats and C-reactive protein in pregnant rats. To sum up, oral androstenedione appears not to cause overt hepatotoxicity in pregnant female rats, although it demonstrated modest changes in lipid metabolism that may facilitate the persistence of damaged cells due to a declined tissue repair rate, which in turn favors disease progression [100]. In other words, androstenedione does not cause liver damage; however, it causes modest changes in lipid metabolism that worsen the present liver damage in the human body.

**Figure 6:**
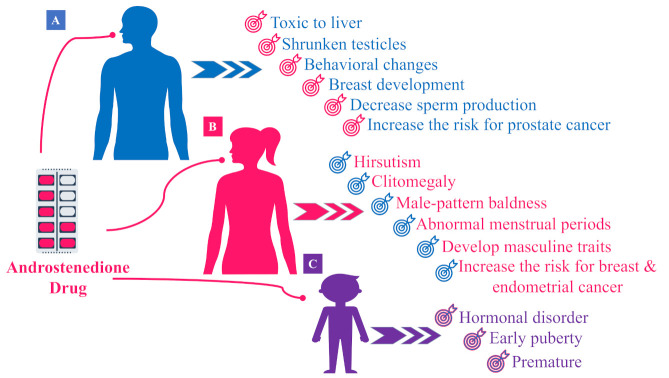
Toxic effects of androstenedione drug supplementation in (**A**) males, (**B**) females, and (**C**) children.

A side effect of androstenedione administration at 100 mg in 10 healthy females produced supraphysiological concentrations of plasma testosterone in healthy women, suggesting that the misuse of anabolic steroids, especially for women, may lead to the development of male characteristics such as male pattern baldness, clitomegaly, voice deepening, hirsutism, abnormal menstrual cycles and abnormal bleeding, blood clots, and metabolic disruption; see Figure 6B. The latter would be very harmful for women, especially those of childbearing age, asides from an increased risk for breast and endometrial cancer [101]. Consequently, drug regulatory authorities should consider classifying androstenedione under the same category as testosterone to control its dispensing and administration. Another study revealed similar results in rats when administering androstenedione (1.0, 5.0, 10.0, or 30.0 mg/kg body weight) for two weeks before mating [102]. They reported that androstenedione at such dose levels significantly increased serum androstenedione, estradiol, and estrone at gestation day 20 in all treated groups. Meanwhile, the serum testosterone concentration was significantly elevated in the 30 mg/kg dose group only, with no observed side effects [102]. The number of female animals showing regular estrous cycles was significantly reduced in the 10.0 and 30.0 mg/kg dose groups. The numbers of implants, viable fetuses, and viable male fetuses were slightly decreased (not statistically significant) in the 30.0 mg/kg androstenedione group. Whether such a hormonal increase can also be observed in fetuses should be monitored for conclusive effects. Finally, androstenedione exposure appears to affect implantation adversely; however, further studies should be conducted with larger numbers of animals or ideally in humans.

Sprando [101] and his colleagues repeated the same experiments but utilizing a larger number of animals and at a higher dose of androstenedione, albeit with slight differences in sampling times. Interestingly, and in contrast to their previous work, serum hormone levels were not significantly affected by androstenedione administration compared to the control group, which could likely be attributed to the rapid metabolism, as the serum hormonal levels were immediately elevated after androstenedione administration and returned subsequently to control values within 24 h [101]. Furthermore, within the two-week pre-mating exposure period, the number of estrous cycles was decreased slightly with the number of animals having irregular estrous cycles to show a slight increase in the androstenedione group (60.0 mg/kg), in contrast to their previous study [102].

Owing to the ambiguous results regarding androstenedione’s effects in female animals, the National Toxicology Program comprehensively investigated the sub- and chronic toxicity and carcinogenicity in both male and female rats and mice at a broad range of androstenedione dose levels (1, 5, 10, 20, 50 mg/kg/day) for 14 weeks in order to gain more conclusive results [94]. Results revealed that androstenedione did not reveal any dose-limiting toxicity in either female or male mice and rats. Although no genetic toxicity was observed in the 14-week study, except for an equal response in the peripheral blood micronucleus test that was observed with androstenedione (50 mg/kg/day) in female mice, it was suggested that there is a potential adverse effect on male fertility and reproductive performance. Such a hypothesis was based on reduced sperm counts in a dose-dependent manner in the rat cauda epididymis by 17%, 20%, and 30% at 10, 20, and 50 mg/kg. Compared to the acute study, the chronic study showed that androstenedione exhibited carcinogenic effects in female and male mice livers, but it may or may not be carcinogenic in rats [94]. Such results should be pursued further, especially given that androstenedione is typically administered over a long-time span as a supplement for athletes.

The side effects of androstenedione related to the increase in testosterone levels in males are still not conclusive, with several reports of elevated serum testosterone and/or estradiol levels [10,67], versus others showing no changes [67]. A consensus was reached that, in most studies, androstenedione was unlikely to provide any anabolic benefit and may even result in adverse health consequences, including sperm count reduction, impotence, and gynecomastia and prostate enlargement, as shown in Figure 6A. Administration to children and adolescents could lead to hormonal effects similar to those obtained upon the cessation of bone growth in adults, early puberty, and pre-matures [101]; see Figure 6C.

The controversy of the toxicity results regarding androstenedione may be explained by several factors: (1) time of serum selection—androstenedione has a high metabolic rate and may be converted into estrogens directly, (2) sex differences, and (3) effects of the surrounding environment, such as residual drug mixtures and pH. For instance, ketoprofen in combination with androstenedione and estrone proved to be synergistically toxic. In addition, the change in pH has a significant effect on the toxicity of androstenedione, suggesting that androstenedione should not be combined with drugs that affect the pH of the body, such as aspirin, or avoided in the case of lung or kidney disorders [100].

## 9. Conclusions

Androgens or steroids should be regulated with extensive medical supervision, specifically androstenedione or 4-Androstene-3-17-dione (4A) and its derivatives. Their metabolites affect humans and non-humans, i.e., fungi, animals, rodents. Moreover, athletes only consider the increase in testosterone levels and bone maturation, disregarding the other known and unknown consequences of the administration of such supplements. Additionally, several of these positive effects are not yet fully scientifically proven. It was reported that androstenedione is carcinogenic in male and female mice, with a limited number of available androstenedione carcinogenic data, warranting more studies to provide a broader view of the dosage limit to reduce, or prevent, such toxic effects. Obviously, many toxic effects occur due to the supplementation of androstenedione among males, females, and children in comparison to its benefits. This review aimed to provide detailed insights into androstenedione’s consumption, metabolism, health effects, and toxicity. It is expected that with more research data available regarding androstenedione drug supplementation, greater control of the useful dose for human health will become possible.
